# Predictors of Disengagement and Symptom Improvement Among Adults With Depression Enrolled in Talkspace, a Technology-Mediated Psychotherapy Platform: Naturalistic Observational Study

**DOI:** 10.2196/36521

**Published:** 2022-06-22

**Authors:** Doyanne Darnell, Michael D Pullmann, Thomas D Hull, Shiyu Chen, Patricia Areán

**Affiliations:** 1 Department of Psychiatry & Behavioral Sciences University of Washington Seattle, WA United States; 2 Talkspace Seattle, WA United States

**Keywords:** depression, psychotherapy, disengagement, internet, web-based, technology-mediated psychotherapy

## Abstract

**Background:**

Depression is a common psychiatric condition with an estimated lifetime prevalence for major depression of 16.6% in the US adult population and is effectively treated through psychotherapy. The widespread availability of the internet and personal devices such as smartphones are changing the landscape of delivery of psychotherapy; however, little is known about whether and for whom this type of therapy is beneficial, and whether having synchronous video-based sessions provides additional benefits to clients above and beyond messaging-based therapy.

**Objective:**

This study examined the outcomes associated with the use of a digital platform (Talkspace) for technology-mediated psychotherapy. We examined the duration of client engagement in therapy and client depression score trajectories over 16 weeks. We explored the association of client characteristics, therapist characteristics, and service plan type with time-to-disengagement and trajectories of change in depression scores.

**Methods:**

This naturalistic observational study assessed data collected routinely by the platform between January 2016 and January 2018 and examined psychotherapy outcomes among a large representative sample of adult clients with clinically significant depression. Treatment disengagement was defined as a lack of client-initiated communication for more than 4 weeks. Clients completed the Patient Health Questionnaire-8 item (PHQ-8) at intake and every 3 weeks via an in-app survey. Cox regression analysis was used to examine the time until and predictors of disengagement. Changes in depression scores and predictors of change over time were examined using mixed-effects regression.

**Results:**

The study included 5890 clients and 1271 therapists. Client scores on the PHQ-8 declined over time, with the average client improving from a score of 15 to below the clinical cutoff of 10 by week 6. At the same time point, 37% of clients had disengaged from the therapy. When combined into a final Cox regression model, those who were more likely to disengage were clients aged 18 to 25 years versus those aged ≥50 years (odds ratio [OR] 0.82, 95% CI 0.74-0.9; *P*<.001), had higher education (OR 1.14, 95% CI 1.06-1.22; *P*<.001), had been in therapy before (OR 1.09, 95% CI 1.02-1.17; *P*=.01), and were living with a partner but unmarried versus single (OR 1.14, 95% CI 1.02-1.27; *P*=.02). Having a therapist with >10 years of experience was related to lower odds of disengagement (OR 0.87, 95% CI 0.8-0.94; *P*=.01). When combined into a final regression model predicting improvement in depression scores over time, clients showing more improvement were those with an associate’s degree or higher (linear estimate=−0.07, *P*=.002) and higher intake PHQ-8 scores (estimate=3.73, *P*<.001). There were no differences based on the plan type.

**Conclusions:**

Our findings add to the growing literature showing the benefits of technology-mediated psychotherapy over a relatively brief period (16 weeks).

## Introduction

### Background

Depression is a common psychiatric condition with an estimated lifetime prevalence of major depression of 16.6% and a previous year prevalence of a major depressive episode of 7.8% to 8.6% among the US adult population [1,2]. Psychotherapy is an effective treatment for depression [3] and is often preferred to medication [4]; however, clients face numerous challenges in obtaining what has traditionally been face-to-face in-person treatment, such as the need to travel to a psychotherapist’s office and potential stigma associated with accessing this care [5]. The widespread availability of the internet and personal devices such as smartphones is changing the landscape of delivery of psychotherapy, allowing consumers to access care more flexibly and without the need to go to a clinic [6]. These opportunities continue to grow in the wake of the COVID-19 pandemic, and the resultant increase in web-based health care is initiated in response to social distancing policies [7].

Numerous technology platforms are now available that allow clients to flexibly communicate with a psychotherapist via text messages, voice, or videoconferencing software; this is known as technology-mediated psychotherapy [6]. A common distinction made regarding technology-mediated psychotherapy is whether it is conducted in an asynchronous or synchronous fashion. Asynchronous refers to communication sent and received by therapists and clients at different times, such as a therapist sending a client an email with a homework assignment and then sending feedback on the assignment once completed. Synchronous refers to communication sent and received by therapists and clients at the same time, such as telephone conversations or a web-based chat session.

### Prior Work

Over the past 20 years, numerous clinical trials of technology-mediated synchronous and asynchronous psychotherapy have been conducted, most of which have evaluated structured cognitive-behavioral approaches. Findings from meta-analyses based on trials conducted in English of psychotherapy for depression by videoconference or telephone (synchronous) indicate that these forms of therapy are similarly effective to face-to-face in-person treatment and with similar (~80%), if not better, completion rates [8-10]. These meta-analyses reflect diverse samples recruited from a variety of settings (eg, veterans, primary care, HIV clinics, outpatient psychotherapy clinics, and community samples).

Fewer studies on the effectiveness of text- or chat-based therapy (whether synchronous or asynchronous) have been conducted, and the findings have been mixed. One clinical trial evaluating asynchronous cognitive behavior therapy (CBT)–oriented psychotherapy for depression among adults in Sweden assessed the benefit of an 8-week email-based therapy, indicating that email therapy was associated with pre-post benefit but no difference in treatment outcomes as compared with a guided 8-week self-help web-based program or waitlist-control at 6 months [11]. The rates of client completion of the intervention were similarly high between the groups (96% completed the email-based therapy and 93% completed the self-help). Another trial using a web-based platform for chat or text-based psychotherapy found that adult primary care patients in the United Kingdom assigned to a web-based CBT-oriented therapy for 16 weeks experienced greater improvement in depression symptoms than a waitlist or usual primary care comparison group over 8 months [12]. Analyses were based on an intent-to-treat sample, of which a large proportion (48%) did not complete the therapy as intended, according to the therapist’s report.

Few studies on technology-mediated psychotherapy have examined how client demographics moderate outcomes or therapy completion. A secondary exploratory analysis of the United Kingdom chat or text-based CBT trial [12] examined moderators of treatment outcome at the 4-month follow-up and found that depression outcomes were better for patients with higher baseline depression severity [13]. In addition, being separated, divorced, or widowed as opposed to being single or married was associated with greater treatment outcomes for those in the CBT condition; age and education level were not significant moderators. Findings from a meta-analysis of a broader array of technology-mediated psychological interventions targeting various mental health diagnoses suggest that client age may be a relevant moderator of outcome; in particular, adults aged 19 to 39 years may experience greater benefits than older or younger clients [14].

Little is known about the effects of technology-mediated psychotherapy delivered routinely, for whom this approach may be beneficial, or whether there is differential effectiveness based on asynchronous versus synchronous delivery. In addition, to date, no studies have compared asynchronous and synchronous technology-mediated psychotherapy. This study aims to fill these gaps by studying outcomes associated with the use of a digital platform (Talkspace) that facilitates technology-mediated psychotherapy either asynchronously or synchronously among adult clients experiencing depression symptoms. Specifically, the platform is a desktop computer and mobile smartphone app that allows 24/7 communication via a secure interactive text chat and voice or video messages with a licensed psychotherapist for a fee [15]. Clients may pay for a plan with optional synchronous 1-hour sessions conducted through chat, telephone, or videoconferencing that can be either 1 or 4 times per month. Several observational studies with selected samples of Talkspace users suggest that the use of the asynchronous text-based psychotherapy plan is associated with improvement in depression symptoms over 12 to 16 weeks of treatment; however, none of these studies examined predictors of these outcomes and for whom the therapy may be most beneficial [15-18].

### Objective

This naturalistic observational study harnesses data collected routinely by the platform and examines psychotherapy outcomes among a large representative sample of clients with clinically significant depression. We examined how long clients remained in therapy before disengaging from the application after their initial enrollment as well as client trajectories of depression scores over 16 weeks after enrollment. In addition, we explored the association between client characteristics, therapist characteristics, and the type of service plan with time-to-disengagement and trajectories of change in depression scores.

## Methods

### Design and Procedure

This study included longitudinal and observational research with clients who signed up for Talkspace between January 2016 and January 2018 and their therapists using data collected routinely as part of the service. Clients accessed the service through an internet search and either paid out-of-pocket or submitted costs to a private insurance plan for out-of-network reimbursement. The signup process includes a brief intake with a consultation therapist who helps the client select their desired plan (eg, messaging only, monthly video, or weekly video plans) and records the client’s therapist preferences, presenting complaints, and demographics. This information is used by the service to identify and offer the client a choice among 3 therapists that most closely match the client’s preferences, are licensed in the client’s state of residence, and have a successful history of treating conditions the client is reporting. After therapist selection, the client is offered a baseline symptom assessment and can then message their therapist as often as they like. Therapists respond to client messages within 24 hours, gather information for a diagnosis, explain the frame of the medium, and conduct informed consent procedures, after which therapy proceeds 5 days a week.

Therapists encounter the platform through internet searches, professional organizations, and peer contacts. All prospective therapists completed an application process to verify the state licensure, training and degree type, professional insurance, background checks, and confirmation of meeting the National Certification of Quality Assurance standards. Following verification, therapists completed an orientation to the platform, received Health Insurance Portability and Accountability Act, privacy, and security training on the proper use of technological media for delivering care, and took on a small number of training cases with supervision for 30 days before beginning their practice on the platform. Engagement and outcome metrics were monitored after the training phase for quality assurance along with a random peer-review process by other therapists on the platform. The service does not prescribe a specific approach to therapy, and while most therapists (61%) report practicing from a cognitive-behavioral orientation, therapists from many traditions are represented on the platform.

### Participants

The participants in this study were clients of the Talkspace service. Inclusion criteria was age 18 years and older, with clinically significant depression as indicated by having a primary diagnosis of depression or a depression-related disorder, and a score of 10 or greater on the 8-item version of the Patient Health Questionnaire (PHQ-8) administered at intake for the service. In addition, given the requirements of the service, participants were English language literate, had internet access, and were able to use mobile or desktop applications for the service. Exclusion criteria for the use of the service were indications during the intake with the consultation therapist, or at any point when working with the treating therapist, of any schizophrenia spectrum and psychotic disorder, or any diagnosis with psychotic features, or any condition requiring hospitalization, or suicidal thoughts or behavior sufficient to be marked a *Yes* on any of questions 3 through 6 on the Columbia Suicide Severity Rating Scale Lifetime-Recent Screen [19] that would require a more intensive level of care than can be offered through an outpatient service.

### Measures and Variables

#### Client Characteristics and Demographics

As part of signing up for the Talkspace service, clients provide their age range (19-25, 26-35, 36-49, 50+ years), gender (female, male, gender queer, transgender male, transgender female, gender variant, other), education level (high school, bachelor’s degree or higher), whether they had ever been in therapy before, and their marital status (single, married, living with a partner, divorced, separated, and widowed).

#### Depression-Related Diagnosis

Clients were assigned a psychiatric diagnosis according to the 5th edition of the Diagnostic and Statistical Manual of Mental Disorders by their matched therapist, which may be assessed through a mix of diagnostic interviewing, often by video, and delivery of standardized symptom assessments. Clients were included in the study if they had a primary diagnosis of a depression-related disorder (eg, major depressive disorder, adjustment disorder with depressed mood, dysthymic disorder, or other mood disorders).

#### Therapist Characteristics

Therapists provide information about demographics and professional experiences when they apply to join a service network. Therapists can select female, male, gender queer, transgender male, transgender female, gender variant, or other gender. Therapists indicate years of postlicensure experience as psychotherapists (coded as <5 years, 5-10 years, and >10 years) and areas of expertise (for this study, dichotomously coded as whether therapists indicated expertise in treating depression).

#### Psychotherapy Disengagement

Disengagement from psychotherapy was defined for this study by a lack of client-initiated communication via any means using the Talkspace application (eg, text, voice messages, and videoconferencing sessions) for more than 4 weeks after initial enrollment. These data are drawn from administrative records on exchanges with therapists and are passively collected by the application.

#### Depression Symptoms

Clients were asked to complete the PHQ-8 [20] at intake and every 3 weeks via an in-app survey. There was an allowance for completing these with a 1-week buffer before or after the deadline. The PHQ-8 includes 8 items that assess the severity of depression symptoms according to the Diagnostic Statistical Manual, Fourth Edition, criteria, with the exception of suicidality or preoccupation with death. Items were rated on a scale from 0 (not at all) to 3 (nearly every day) and summed to obtain a total score. Higher scores indicate greater severity, and a cutoff score of 10 or higher indicates clinically significant depression. A 5-point difference in scores indicates a clinically meaningful change in depression symptoms [21].

#### Plan Type

Platform clients could opt to purchase one of three service plans: (1) unlimited text, voice, or video messages; (2) unlimited text, voice, or video messages plus once per month 1-hour videoconferencing session; or (3) unlimited text, voice, or video messages plus 4 times per month 1-hour videoconferencing sessions. How often and to what extent clients and therapists communicate varies and is dependent upon the frequency with which clients send messages to their therapist. Therapists are expected to respond to client communications within 24 hours and generally respond within 12 hours, except for therapist days off or other times mutually agreed upon by the therapist and client.

### Plan of Analysis

Using R, we examined descriptive statistics for client and therapist characteristics, depression symptoms at intake or baseline, and service plan type that clients were enrolled in. We examined the missingness of study variables at each point of time as described in the description of our results. For the prediction analysis, we collapsed several categorical variables when the sample size within some of the variable categories was low, which made theoretical sense to do so. We collapsed education into high school education versus associate’s degree or higher. As only 30 participants were widowed, we collapsed this group into the missing category. We conducted 2 sets of mixed effects regression analyses to examine, (1) trajectories of disengagement over 52 weeks and predictors of disengagement and (2) trajectories of depression scores over 16 weeks and predictors of change in depression over time.

#### Predicting Psychotherapy Disengagement Over 52 Weeks After Intake

We examined how long clients were in therapy before disengaging over the course of 52 weeks, following their intake with the platform. Furthermore, a duration of 52 weeks was selected based on visual inspection of the data, showing that most participants had stopped communicating with their therapist for more than 4 weeks by that time. Using R, we conducted mixed-effects Cox regression analyses predicting time until disengagement, with random terms for clinician and client to account for nesting of time within client and client within therapist. Model building followed appropriate procedures for our combined confirmatory and exploratory analyses; predictors were first tested individually to conduct a priori confirmatory hypothesis testing, and those that were significant at *P*<.05 were included in a final, combined exploratory model to examine the overall model structure. Significance was tested using the log-rank test for individual variable models. For categorical variables, simple contrast comparisons were used in which each category was compared against the reference category, as described in the results. To estimate the goodness-of-fit, we computed the proportion of the variance accounted for using Nagelkerke *R*^2^ for each model. We also computed the concordance statistic, which provides the fraction of concordant pairs between the model-predicted and actual data and is equal to the receiver operating characteristic curve. A concordance statistic of 0.50 means that the model is no better than random chance, while a statistic of 1.0 would mean there is a perfect concordance between predicted and actual data. Analyses of the relationship between missing data status on demographic and descriptive variables and time until engagement were conducted separately using Cox regression analyses; cases with missing data were excluded from the primary analyses.

#### Predicting Depression Symptoms Over 16 Weeks After Intake

We examined depression symptoms based on PHQ-8 scores over the course of 16 weeks following the client’s service intake. In addition, a duration of 16 weeks was selected based on visual inspection of the data showing that most of the change in scores occurred during the first 16 weeks and missing data were substantial at that point, which is likely primarily owing to high rates of client disengagement from therapy by 16 weeks. Using R, mixed-effects regressions with random terms for therapist and client were computed to adjust standard errors owing to nesting of time within client and client within therapist. The covariance matrix was specified to be unstructured. As described below, the rates of missing data were high for the dependent variable owing to ending treatment or incomplete measures. Missing data were determined to be not missing at random, based on the empirical analyses described below and theoretical reasons. It is highly likely that those who did not complete measures were due to unmeasured reasons, such as treatment attitudes, motivation, and emotional distress or wellness. Therefore, full information maximum likelihood estimation was used for model testing and parameter estimation (to maximize all available data), and variables associated with missing outcome data were included in the final models to statistically adjust for missingness to the greatest extent possible. Findings may only be inferred to the population of clients who completed measures while receiving treatment. Model building followed standard procedures [22]. Model fit was tested by computing the Akaike information criterion, Bayesian information criterion, and 2-Log Likelihood deviance statistics. The models tested linear, quadratic, and cubic time trends for changes in the PHQ-8 scores over time. The best-fitting models included the linear and quadratic time trends. Then, each potential predictor, including client and therapist characteristics, was entered into individual models, and interaction terms for linear and quadratic time were tested using model fit deviance statistics, with each more complex model tested against simpler models, and all available data were used for each computation. All variables and interaction terms from models that were statistically significant at *P*<.05 were then entered into a final omnibus model. We ran two types of *R*^2^ for each model: the conditional *R*^2^ provides the total proportion of variance accounted for, including fixed and random terms, while the marginal *R*^2^ provides the proportion of variance accounted for by fixed terms only.

### Ethics Approval

As part of the terms of service agreement, clients agreed that their anonymized data may be used for research purposes. Considering the data were completely anonymized, this study was exempted from institutional review board approval by the institutional review board of the University of Washington.

## Results

### Sample Characteristics

The study included 5890 clients and 1271 therapists (Table 1). The participants were primarily female (4504/5890, 76.5%), 26 to 35 years of age (3061/5890, 52%), had a bachelor’s degree (3706/5890, 62.9%), were single (3122/5890, 52.8%), and had been in therapy before (4004/5890, 68%). The average PHQ-8 score was 15.2 (SD 3.9). The therapists were primarily female (1114/1271, 87.6%), with more than 5 years of experience (1017/1271, 80%), and close to half endorsed having depression-specific expertise (591/1271, 46.5%). Clients predominantly signed up for a messaging-only Talkspace plan (5389/1271, 91.5%). There were no missing data for client disengagement from therapy; however, we observed modest amounts of missing data for other client and therapist variables (ranging from 0.3% to 13.6%, see Table 1). Missing data analyses found that missing data on the following variables were not significantly associated with the length of time until disengagement: client’s gender, client’s first time in therapy, or therapist’s years of experience. Client age and education were significantly associated, such that those who had missing data on these variables were more likely to disengage (age odds ratio [OR] 1.46, 95% CI 1.15-1.89, *P*=.002; education OR 1.10, 95% CI 1.02-1.19, *P*=.013).

**Table 1:** Client and therapist characteristics.

Variable		Value
Client: total, n (%)		5890 (100)
**Client age (years), n (%)**		
	18-25	1328 (22.5)
	26-35	3061 (52)
	36-49	1182 (20.1)
	>50	250 (4.2)
	Missing	69 (1.2)
**Client gender, n (%)**		
	Female	4505 (76.5)
	Other	5 (0.1)
	Queer	9 (0.2)
	Variant	5 (0.1)
	Male	1317 (22.4)
	Transgender female	3 (0.1)
	Transgender male	7 (0.1)
	Missing	39 (0.7)
**Client education, n (%)**		
	High school	1327 (22.5)
	Some college	14 (0.2)
	Associate’s degree	6 (0.1)
	Bachelor’s degree or higher	3706 (62.9)
	Master’s degree	21 (0.4)
	Doctoral degree	5 (0.1)
	Professional degree	8 (0.1)
	Missing	803 (13.6)
**Client marital status, n (%)**		
	Divorced	328 (5.6)
	Living with a partner	497 (8.4)
	Married	1663 (28.2)
	Separated	142 (2.4)
	Single	3112 (52.8)
	Widowed	30 (0.5)
	Missing	118 (2.0)
**Client first time in therapy**		
	No, n (%)	4004 (68)
	Yes, n (%)	1346 (22.9)
	Missing, n (%)	540 (9.2)
	Baseline PHQ-8^a^, mean (SD)	15.2 (3.9)
**Talkspace plan type**		
	Text only, n (%)	5389 (91.5)
	1-month video, n (%)	411 (7)
	4-month video, n (%)	90 (1.5)
	Therapist, total (N)	1271 (100)
**Therapist gender, n (%)**		
	Male	157 (12.4)
	Female	1114 (87.6)
**Therapist years of experience, n (%)**		
	<5	250 (19.7)
	5-10	551 (43.4)
	Missing	4 (0.3)
Therapist with depression expertise, n (%)		591 (46.5)

^a^PHQ-8: Patient Health Questionnaire-8 item.

### Predicting Psychotherapy Disengagement Over 52 Weeks Post Intake

**Figure 1:**
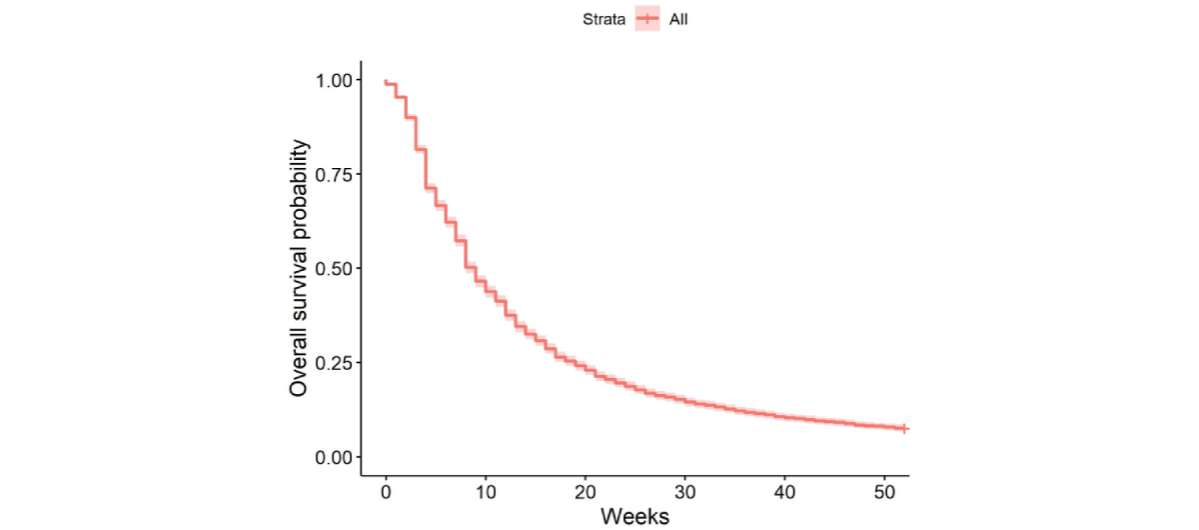
Time until disengagement.

**Figure 2:**
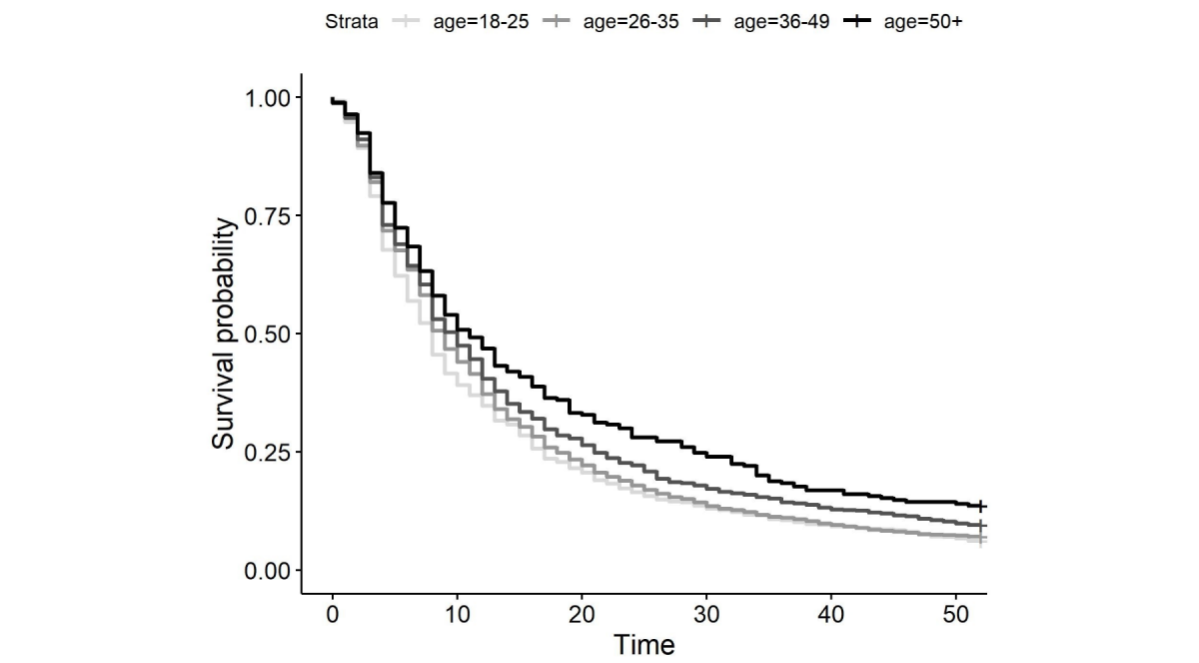
Time until disengagement stratified by age group.

By week 6, 37% of the clients had disengaged from therapy. Half of the sample disengaged from therapy by week 9 and by week 52, nearly all clients had disengaged (n=5441, 92.4%; Figure 1). Table 2 displays the single-variable predictor models for all the variables that were significantly associated with disengagement. When combined into a final Cox regression model, significant variables included client age, education, whether the client had been in therapy before, marital status, and years of experience as a therapist (Table 3). Compared with those aged 18 to 25 years, those aged 36 to 49 years had 18.9% lower odds of disengaging at any point in time (OR 0.82, 95% CI 0.74-0.9; *P*<.001; Figure 2) and those aged >50 years had 30.1% lower odds of disengaging (OR 0.70, 95% CI 0.59-0.83; *P*<.001). Those with higher education had 13.5% greater odds of disengaging than those with lower education (OR 1.14, 95% CI 1.06-1.22; *P*<.001), and those who were in therapy for the first time had 8.9% greater odds of disengaging (OR 1.09, 95% CI 1.02-1.17; *P*=.01). Participants who were living with a partner had 13.9% greater odds of disengaging as compared with those who were single (OR 1.14, 95% CI 1.02-1.27; *P*=.02); there were no significant differences between those who were single and those who were married, separated, or divorced. Finally, clients with therapists who had more than 10 years of experience had 13.2% lower odds of disengaging (OR 0.87, 95% CI 0.8-0.94; *P*=.01). Although significant in the single-variable predictor model, there were no significant differences in the likelihood of disengagement for clients based on the therapist endorsement of depression-specific expertise in the combined Cox regression model. Plan type, intake PHQ-8 score, client gender, and therapist gender were not included in the final model, as these variables were not significant in the single-variable predictor models. Model fit statistics revealed a very low proportion of variance accounted for and concordance across all models (Tables 3 and 4). The proportion of variance accounted for in the final model was only 1.8%, and the concordance statistic of 0.54 indicated that the model predicted the length of time until disengagement only slightly better than chance.

**Table 2:** Significant single-variable predictor Cox regression models testing time until treatment disengagement^a^.

		Median (weeks)	OR^b^ (95% CI)	*P* value	Log rank		*P* value	
**Age (years)**						33.78		<.001
	18-25	8	Ref	Ref				
	26-35	9	0.93 (0.87-0.99)	.035				
	36-49	10	0.83 (0.77-0.91)	<.001				
	>50	11	0.71 (0.61-0.82)	<.001				
**Education**						17.24		<.001
	High school	9	Ref	Ref				
	Associate’s degree or higher	8	1.15 (1.08-1.22)	<.001				
**First time in treatment**						11.37		<.001
	No	9	Ref	Ref				
	Yes	8	1.12 (1.05-1.19)	<.001				
**Marital status**						15.31		.004
	Single	9	Ref	Ref				
	Living with partner	8	1.18 (1.07-1.3)	.001				
	Married	9	0.96 (0.91-1.03)	.22				
	Separated	10	0.98 (0.82-1.17)	.83				
	Divorced	9	0.95 (0.84-1.06)	.37				
**Therapist experience**						16.45		<.001
	<5 years	8	Ref	Ref				
	5-10 years	9	0.93 (0.86-1)	.07				
	>10 years	9	0.86 (0.8-0.93)	.002				
**Therapist with depression-specific expertise**						7.97		.004
	Not endorsed	8	Ref	Ref				
	Endorsed	9	0.92 (0.88-0.98)	.03				

^a^Client n=5890, therapist n=1271. Variables that were tested and were not significant included plan type, intake PHQ-8 score, client gender, and therapist gender. Widowed data were collapsed with missing marital status data. Disengagement from treatment was defined as >4 weeks without any client-initiated communication to the therapist, such as texting, video exchanges, images, or audio clips. The modeling included up to 52 weeks of therapy.

^b^OR: odds ratio.

**Table 3:** Model fit statistics for individual and final combined nested Cox regression model predicting time until treatment disengagement^a^.

			Nagelkerke *R*^2^	Concordance
**Individual models**				
	Age	0.0065		0.52
	Education	0.0036		0.52
	First time in therapy	0.0022		0.51
	Marital status	0.0028		0.51
	Therapist experience	0.0030		0.51
	Therapist with depression-specific expertise	0.0015		0.51
Final model			0.018	0.54

^a^Disengagement from treatment was defined as >4 weeks without any client-initiated communication to the therapist, such as texting, video exchanges, images, or audio clips. The modeling included up to 52 weeks of therapy.

**Table 4:** Final combined nested Cox regression model predicting time until treatment disengagement^a^.

			Coefficient		OR^b^ (95% CI)		*P* value
**Age (years; ref=18-25)**							
	26-35	−0.05		0.96 (0.88-1.03)		.27	
	36-49	−0.20		0.82 (0.74-0.9)		<.001	
	>50	−0.36		0.7 (0.59-0.83)		<.001	
**Education (ref=high school)**							
	Associate’s or bachelor’s degree or higher	0.13		1.14 (1.06-1.22)		<.001	
	First time in treatment	0.09		1.09 (1.02-1.17)		.011	
**Marital status (ref=single)**							
	Living with a partner	0.13		1.14 (1.02-1.27)		.019	
	Married	0.03		1.03 (0.95-1.1)		.48	
	Separated	0.006		1.01 (0.82-1.23)		.95	
	Divorced	0.04		1.04 (0.91-1.2)		.55	
**Therapist experience (years; ref=<5)**							
	5-10	−0.09		0.92 (0.84-0.99)		.06	
	>10	−0.14		0.87 (0.8-0.94)		.01	
	Therapist with depression-specific expertise	−0.06		0.94 (0.89-1)		.14	

^a^Disengagement from treatment was defined as >4 weeks without any client-initiated communication to the therapist, such as texting, video exchanges, images, or audio clips. The modeling included up to 52 weeks of therapy. Predictor variables that were significant in simpler models were also included.

^b^OR: odds ratio.

### Predicting Depression Symptoms Over 16 Weeks Post Intake

There were high levels of missingness in the PHQ-8 over the 16 weeks after the intake observation period. Of the 5890 clients, 3447 (58.5%) completed a 3-week survey, 1978 (33.6%) completed a 6-week survey, 1165 (19.8%) completed a 9-week survey, 724 (12.3%) completed a 12-week survey, and 506 (8.6%) completed a 15-week survey. When comparing those with at least one follow-up time point to those without, there were no differences in the rates of missing data on the basis of age, education level, history of therapy, gender, or PHQ-8 score. Those who did not have any follow-up PHQ-8 were more likely to be divorced (8.1% with follow-up data vs 9.6% missing all follow-up) or living with a partner (8.1% vs 9.6%), less likely to be married (30.2% vs 27.1%), more likely to have a video plan (7.9% vs 9.4%), and less likely to have a messaging-only plan (90.6% vs 92.1%).

**Figure 3:**
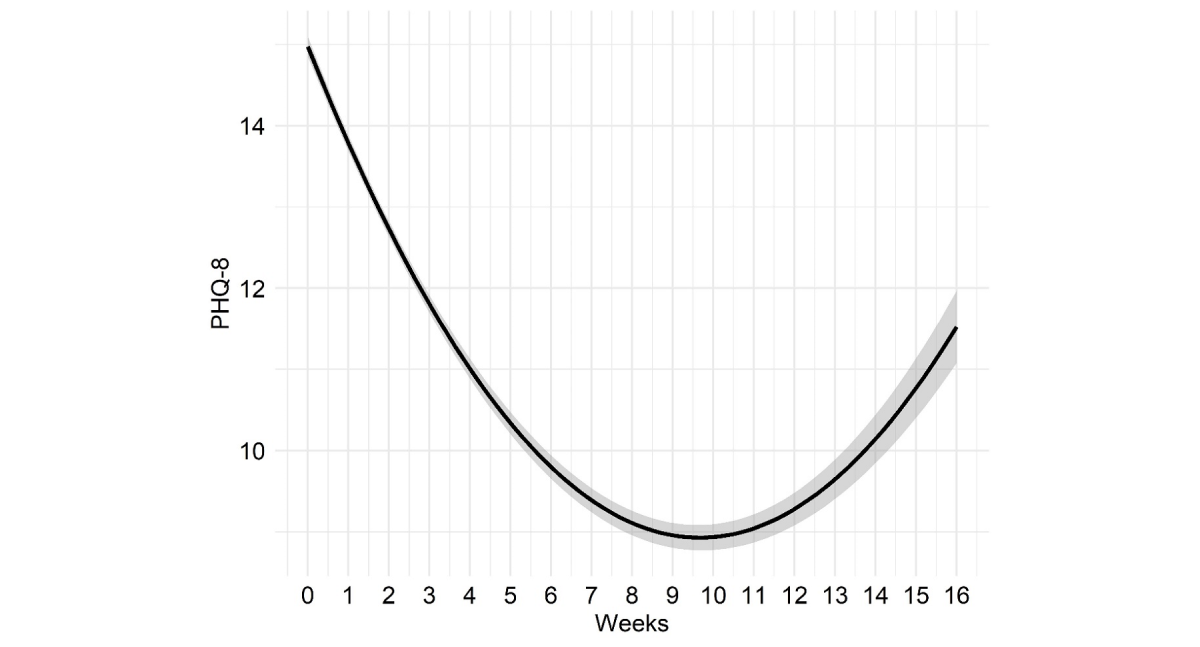
Model estimated change on the Patient Health Questionnaire-8 item (PHQ-8) over 16 weeks with SE shading.

The best-fitting model for change over time in the PHQ-8 is shown in Figure 3, illustrating a curvilinear change in PHQ-8 scores during the first 16 weeks of treatment (quadratic -2LL_5_=−41,432, deviance=399, *P*<.001), therefore all subsequent models included linear and quadratic change variables. The model-derived average PHQ-8 score at intake was 14.94 (SE 0.08), with a linear slope decreasing an average of −1.25 points (SE 0.03) between the first and second week and flattening by an additional 0.06 points (SE 0.002) for each subsequent week. Client scores on the PHQ-8 declined over time, dropping below the clinical cut-off from a score of 15 to 10 by week 6.

Table 5 displays models using single variables to predict change over time and found that client education level and age significantly predicted intake PHQ-8 score and linear change over time (education level linear – 2LL_2_ deviance=25, *P*<.001; age linear 2LL_6_ deviance=9, *P*=.006). Clients’ PHQ-8 score at intake, and whether it was their first time in therapy, significantly predicted linear and quadratic change over time in the PHQ-8 score (first PHQ-8 score linear -2LL_2_ deviance=1991, *P*<.001, quadratic -2LL_1_ deviance=99, *P*<.001; first time in therapy linear -2LL_2_ deviance=12, *P*<.001, quadratic -2LL_1_ deviance=9, *P*<.001). Variables that were not significantly associated with PHQ-8 scores over time included plan type, client gender, therapist gender, therapist years of experience, and therapist endorsing depression-specific expertise. Model fit statistics are displayed in Table 6, demonstrating that, unsurprisingly, the model incorporating intake PHQ-8 score accounted for the largest amount of variance in the model.

**Figure 4:**
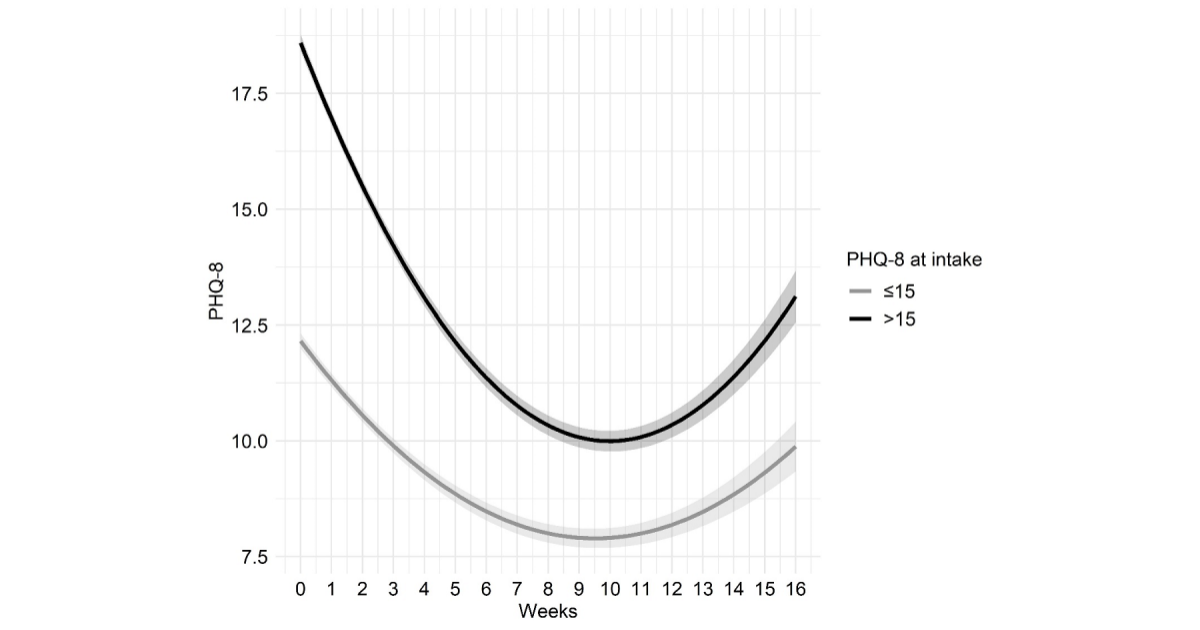
Median split Patient Health Questionnaire-8 item (PHQ-8) score at intake and change over time with SE shading.

The final model explained 51.1% of the variance in PHQ-8 score change. This model included predictor variables that had significant relationships with the intercept and linear or quadratic change in PHQ-8 scores over time, on the basis of single variable modeling (see Table 7). Significant predictors were as follows: those with an associate’s degree or higher improved more quickly based on their sharper linear slope (linear estimate=−0.07, *P*=.002). Higher intake PHQ-8 scores, displayed as a median split in Figure 4, were associated with a larger PHQ-8 intercept (estimate=3.73, *P*<.001), faster linear improvement (estimate=−0.50, *P*<.001) and faster flattening (quadratic estimate=0.03, *P*<.001). There were no differences in the PHQ-8 intercept on whether it was the client’s first time in therapy; however, first-time clients had faster linear improvement (linear estimate=−0.37, *P*<.001) and faster flattening (quadratic estimate=0.03, *P*<.001). Age was not significant after controlling for other variables.

**Table 5:** Significant single-variable mixed effects models predicting Patient Health Questionnaire-8 item (PHQ-8) score change^a^.

			Estimate		SE		*P* value
**Education (ref=high school)**							
	Intercept	15.66		0.13		<.001	
	Week	−1.22		0.03		<.001	
	Week^2^	0.06		0.002		<.001	
	Associate’s or bachelor’s degree or higher	−0.90		0.15		<.001	
	Associate’s degree or higher × week	−0.02		0.02		.34	
**Age (ref=18-25 years)**							
	Intercept	15.26		0.13		<.001	
	Week	−1.24		0.03		<.001	
	Week^2^	0.06		0.002		<.001	
	26-35	−0.49		0.14		<.001	
	36-49	−0.14		0.17		.41	
	>50	−0.42		0.29		.14	
	26-35 × week	−0.01		0.03		.67	
	36-49 × week	0.005		0.03		.87	
	>50 × week	0.05		0.05		.27	
**Intake PHQ-8 score**							
	Intercept	15.07		0.06		<.001	
	Week	−1.28		0.02		<.001	
	Week^2^	0.07		0.002		<.001	
	PHQ-8	3.75		0.05		<.001	
	PHQ-8 × week	−0.49		0.02		<.001	
	PHQ-8 × week^2^	0.03		0.002		<.001	
**First time in therapy**							
	Intercept	15.04		0.09		<.001	
	Week	−1.17		0.03		<.001	
	Week ^2^	0.06		0.003		<.001	
	First time in therapy	−0.20		0.15		.17	
	First time in therapy × week	−0.30		0.07		<.001	
	First time in therapy × week^2^	0.02		0.005		<.001	

^a^Variables that were tested and were not significant on the basis of model fit statistics included client gender, marital status, plan type, therapist gender, therapist years of experience, and therapist endorsing depression-specific expertise.

**Table 6:** Model fit statistics predicting Patient Health Questionnaire-8 item (PHQ-8) score change.

			AIC^a^		BIC^b^		−2LL^c^		df		Conditional *R*^2^		Marginal *R*^2^
**Individual models**													
	Education	66,153.2		66,204.5		−33,069.6		7		0.279		0.180	
	Age	66,082.0		66,162.7		−33,030		11		0.271		0.179	
	Intake PHQ-8 score	62,024.4		62,083.0		−31,004.2		8		0.510		0.460	
	First time in therapy	62,877.0		62,935.3		−31,430.5		8		0.278		0.182	
Final combined model			64,112.8		64,252.3		−32,037.4		19		0.511		0.455

^a^AIC: Akaike Information Criterion.

^b^BIC: Bayesian Information Criterion.

^c^−2LL: −2 Log Likelihood.

**Table 7:** Final combined mixed effects model predicting Patient Health Questionnaire (PHQ-8) score change^a^.

			Estimate		SE		*P* value
Intercept			15.36		0.14		<.001
Week			−1.10		0.04		<.001
Week^2^			0.06		0.002		<.001
Associate’s or bachelor’s degree or higher (ref=high school)			−0.18		0.12		.120
Associate’s degree or higher × week (ref=high school)			−0.07		0.02		.002
**Age (years, ref=18-25)**							
	26-35	−0.16		0.13		.20	
	36-49	−0.10		0.16		.52	
	>50	−0.41		0.26		.12	
**Age (years, ref=18-25) × week**							
	26-35	−0.03		0.02		.15	
	36-49	−0.01		0.03		.76	
	>50	0.04		0.04		.32	
Intake PHQ-8 score			3.73		0.06		<.001
Intake PHQ-8 score × week			−0.50		0.03		<.001
Intake PHQ-8 score × week^2^			0.03		0.002		<.001
First time in therapy			−0.07		0.13		.58
First time in therapy × week			−0.30		0.06		<.001
First time in therapy × week^2^			0.03		0.005		<.001

^a^Predictor and predictor × time interaction variables that were significant in simpler models were included. The intake PHQ-8 scores were grand mean-centered.

## Discussion

### Principal Findings

This is the first population-based study of treatment engagement, subsequent outcomes, and predictors of outcomes in the routine delivery of technology-mediated psychotherapy focused on adult clients experiencing clinically significant levels of depression. Our findings indicate that technology-mediated psychotherapy may be helpful in reducing symptoms of depression. On average, client scores on the PHQ-8 declined over time, dropping below the clinical cut-off from a score of 15 to 10 by week 6. At the same time point, 37% of clients had disengaged from the therapy. There were no differences in the length of treatment engagement or speed of improvement based on whether clients opted for a plan that included synchronous video chat (either once or 4 times per month) in addition to the asynchronous messaging plan.

Findings from our analyses predicting symptom change and disengagement suggest that the degree to which the therapy was helpful did not depend on the client or therapist characteristics included in the study; however, how quickly clients decided to end the therapy did, though this effect was very small. One exception was education level. Completing formal education beyond high school was related to a sharper decrease in PHQ-8 scores and a shorter length of time before disengagement, which may be interpreted as clients with higher education being a group that is particularly likely to benefit quickly from technology-mediated psychotherapy and, therefore, end it sooner. Consistent with this premise, there is some evidence that individuals with more years of formal education are less likely to end therapy prematurely [23]. Clients who were in therapy for the first time were also more likely to improve faster. While this might be associated with less severe depressive symptoms, this result was true even after controlling for baseline depression scores. It may be that those repeatedly seeking treatment have symptoms that are more chronic, even if they are of equal severity to those seeking treatment for the first time.

The fact that specific age categories and gender were not significantly associated with depressive symptom change is consistent with a study examining moderators of outcomes among primary care patients in the United Kingdom randomized to chat-based CBT therapy versus usual care [13]. However, in contrast to our findings, educational attainment did not moderate the relationship between therapy and outcomes in the primary care sample. This is also consistent with a recent meta-regression study examining moderators of outcomes from randomized trials of face-to-face in-person psychotherapy versus a control group among adults with depressive symptoms, in which the authors found no difference between older and younger adults and between men and women [24]. The authors note that there were insufficient studies available for the analysis with a low risk of bias to examine education level as a moderator.

Unfortunately, we did not have data on client race and ethnicity; however, in the meta-regression study mentioned [24], studies focusing on diverse samples did not have better or worse effect sizes than the other studies. This is similar to findings from a previous meta-analysis using a similar methodology, looking at randomized controlled trials in which race and ethnicity data were reported [25]. The authors examined whether the proportion of adults identified as a racial or ethnic minority in the sample was associated with treatment effect size across 56 trials and found no relation. Although psychotherapy appears to be effective across racial and ethnic groups [26], access to and barriers to engagement in psychotherapy are characterized by racial or ethnic disparities [27]. For instance, a large meta-analysis of randomized controlled trials of face-to-face in-person psychotherapy for depression among adults found that studies with a higher proportion of participants identifying with a racial or ethnic minority group had higher rates of early therapy termination, as defined by research teams [28].

In a nationally representative US-based study, people identified as Black or African American, Latinx, and Asian Americans were less likely to engage in mental health outpatient care and indicated financial costs to be a major barrier to accessing services [29]. Other barriers may be related to beliefs that therapy is not likely to be effective, stigma and shame about needing care and being seen accessing it, language barriers, and cultural practices that may not resonate with a Western medical model [27,30-32]. Technology-mediated psychotherapy holds promise for alleviating some of these barriers to psychotherapy, particularly in reducing the need to find a local therapist and attend in-person sessions that may require transportation and additional time to travel to appointments. Although technology-mediated therapy may increase a sense of privacy and confidentiality as clients do not have to present to a clinic or office for care where others may see them, there may be additional barriers to ensuring privacy that have to do with concern over security breaches [33]. Although technology and internet access are expanding rapidly, there are still substantial disparities in access to stable and consistent internet and internet-capable smartphones and computers [34]. Future research can examine the role of race, ethnicity, and the intersection of access to technology-mediated psychotherapy in both outcomes and therapeutic engagement.

Our findings also indicate that the length of engagement in services is weakly associated with the variables included in this study. More research should examine the determinants of engagement and work to operationalize them using feasible methods so that clinical care providers can target their efforts to prevent premature disengagement. While the association is weak, our findings indicate that being younger, in therapy for the first time, unmarried but living with their partner (as compared with single), and being with a therapist with less experience predicted a shorter length of time to disengagement from the therapy. Previous research indicates that younger clients are more likely to prematurely end therapy [23], and that less experienced therapists tend to have higher premature termination rates [23] and more difficulty engaging and retaining clients in therapy [35]. Marital status did not appear to be related to psychotherapy or mental health service disengagement in previous face-to-face psychotherapy studies [23,28,36]. However, a study of chat-based CBT with primary care patients observed that those patients grouped into a category of separated, divorced, or widowed had greater benefit from the therapy than those who were married or living as married and those who were single. The authors determined that the effect seemed to be largely owing to the separated, divorced, or widowed group remaining more symptomatic when waitlisted and obtaining usual care. Understanding the role of marital status in therapeutic gains is complicated by examination of this variable. Owing to the small sample size, categories that seem conceptually similar on the surface may collapse in analyses but may actually represent very distinct groups empirically [37]. For instance, being unmarried and living with a partner may hold a unique meaning for the millennial generation, which comprises a large proportion of our sample who are known to delay marriage [38], and combining these clients with married clients, as was done in the Button et al [13] study, could obscure findings that would emerge if examined separately, as was done in our analysis. Qualitative research may help elucidate the significance of living with a partner, which may be related to a more rapid disengagement from therapy.

Additional research is needed to understand why client sociodemographic factors may influence perceptions of therapy and decisions to end the therapy. However, it is important to note that not all therapeutic disengagement outcomes are negative. In fact, people are known to vary in how quickly their symptoms respond to psychotherapy, and there is evidence to suggest that people leave therapy when they feel they have a good enough level of change [39-41]. A previous study of Talkspace clients suggested that this is often the case. Specifically, in a study of clients experiencing depression or anxiety, one-third of those leaving therapy before 12 weeks of enrollment reported reasons for leaving, with 53% indicating satisfaction with reaching therapeutic goals as the reason [18]. Therefore, engagement in technology-mediated psychotherapy may require shorter engagement times than in-person session-based therapy, potentially because of the ability of clients to access therapists as needed, rather than the typical once a week or every other week schedule.

This study examined disengagement from the initial enrollment in therapy; however, it is possible that clients re-engaged, and there may be observable patterns of disengagement and re-engagement over time among the clientele. In addition, the concept of disengagement does not shed light on the nature of a client’s engagement in therapy. Therapeutic engagement can be defined as all efforts made by clients during the course of treatment (both within and between sessions) toward the achievement of changes (treatment outcomes) [35]. Once a client has disengaged, it is clear that they no longer participate in the therapy; however, if the client is still in therapy, the degree to which they are genuinely engaged in the process with the therapist can vary substantially and is an important variable in treatment outcomes. In an earlier Talkspace study with depressed or anxious clients, greater amounts of communication between clients in therapists based on word counts across text, audio, and video messaging were related to symptom improvement [18]. Research on face-to-face in-person CBT in which the timing of sessions varied suggests that having more frequent sessions in closer proximity to each other versus longer courses of therapy is related to improved outcomes [42]. A benefit of technology-mediated psychotherapy, such as what the Talkspace platform offers, is that clients have more potential to increase the intensity of their therapy and perhaps more quickly benefit from it.

Interestingly, it may not be necessary for clients to meet with their therapist in a more traditional synchronous manner, such as having a face-to-face web-based video-based session, to receive an adequate degree of therapeutic intensity through technology-mediated therapy. In this study, we did not observe a relationship between the type of plan clients opted into and changes in depression scores over time, although some plan types included 1 or 4 synchronous video sessions per month in addition to text and audio or video messaging. In fact, the predominant type of plan clients opted for when they enrolled in the Talkspace platform was asynchronous messaging only (n=5389, 91.5%), although we do not know whether clients opted out of the other plans owing to cost, preference, or both.

### Limitations and Considerations

A key consideration for our findings is the nature of clients enrolled in technology-mediated psychotherapy. The most common client characteristics were being of the millennial generation [38], college educated, single, identifying as female, and having experience with therapy. In addition, as the messaging-only plan was by far the most frequently chosen (91.5%), the study reflects clients willing or preferring to engage in primarily text messaging–based therapy, which may or may not be asynchronous. Men are similarly underrepresented in face-to-face in-person therapy [43] and represent a population that may need additional effort to reach and engage in technology-mediated psychotherapy. The population in our study may represent what could be considered early adopters of the innovation of text-based psychotherapy, which is also consistent with the premise that members of the millennial generation are more generally engaged with internet technology and more comfortable using it in myriad ways [44]. In addition, data were collected before the COVID-19 pandemic, when engaging in technology-mediated health care was novel for most people. Considering social distancing policies to control the spread of COVID-19, technology-mediated health care proliferated, and the business of web-based therapy grew tremendously as the general demand for psychotherapy simultaneously increased [7,45]. The postpandemic population may be different from the population included in our study. In fact, a recent study published with postpandemic Talkspace clients suggests that anxiety is a much more common experience and comorbidity among clients since the pandemic started [46].

Our findings for depressive symptom change do not generalize well past the first 6 weeks of therapy, given that missing data on the PHQ-8 increases substantially after 6 weeks. A known contributing factor to missing data on the PHQ-8 is that clients stop taking the measure after disengaging from the therapy. Our estimates of later time points were based on the trajectory that was observed at the time the clients stopped taking the PHQ-8. Given that it is common for people with greater improvement in earlier phases of therapy to end therapy earlier than those whose symptoms do not remit as quickly [47], it is possible that the overall improvement in depression symptoms is an overestimation of what we would observe if clients continued taking PHQ-8 assessments. There are high rates of missing data, aside from that, which can be attributed to therapy disengagement. Therefore, these results may only be generalized to people who are receiving therapy and who complete the PHQ-8 assessments. In addition, given that we observed that higher intake PHQ-8 scores were predictive of symptom improvement, a likely caveat for our findings is that there is some regression to the mean, a statistical artifact of more extreme-scoring individuals to score more closely to the average on the next assessment. In randomized controlled trials, where regression to the mean is accounted for, it is more common to actually see higher initial depression scores as related to less symptom improvement [48-50]. Like all regression models, our curvilinear time modeling provides an overall average estimate of symptom trajectories and is not able to demonstrate what are likely nonlinear relationships with symptoms and time that would be observed if examined at the individual client level [51]. However, we can say that, on average, we see a trend for clients to do better symptomatically than when they enrolled.

We conducted a naturalistic study using routinely collected data from the Talkspace platform. Therefore, there were limitations in the amount and nature of the data available. First, we did not account for other conditions that clients may be seeking therapy for, or conditions that may moderate therapeutic outcomes. For instance, the presence of a personality disorder is related to premature termination of therapy [23] and is known to impede symptom improvement [52]. The PHQ-8 does not capture suicidal thinking, so we do not have estimates of suicide risk using the item from the PHQ-9 that asks about *thoughts that you would be better off dead, or of hurting yourself*, which is known to be predictive of suicidal behavior and a common question asked as part of routine suicide risk screening procedures [53]. In addition, we do not know what type of therapy is provided by licensed clinicians; however, therapy from various theoretical orientations is known to be effective for depression [54]. The availability of textual data allows for an ample exploration of what is happening in routine psychotherapy in this context. Future research can use the rich data made available through text messages and transcripts made from audio or video interactions to examine the degree to which evidence-based interventions and practices are part of the therapy and how this might be related to disengagement, engagement, and symptom outcomes.

### Conclusions

Technology-mediated psychotherapy is becoming an increasingly popular alternative to in-person psychotherapy. Our findings add to the growing literature showing the benefits of technology-mediated psychotherapy in general and messaging-based therapy in particular. In addition, benefits were observed over a relatively brief period, with a reduction in symptoms observed within 16 weeks of therapy. Some populations may require additional efforts to remain engaged once they enroll, such as in younger adults. Future research is needed to examine how well technology-mediated psychotherapy addresses disparities in access to therapy and opportunities to enhance reach and ensure the effectiveness of this treatment modality across the full spectrum of adults experiencing depression.

## Abbreviations

CBT: cognitive behavior therapy
OR: odds ratio
PHQ: Patient Health Questionnaire
